# Incidence, risk factors and outcomes of new-onset atrial fibrillation in patients with sepsis: a systematic review

**DOI:** 10.1186/s13054-014-0688-5

**Published:** 2014-12-15

**Authors:** Sanne Kuipers, Peter MC Klein Klouwenberg, Olaf L Cremer

**Affiliations:** Department of Intensive Care Medicine, University Medical Center Utrecht, Heidelberglaan 100, 3584 CX Utrecht, The Netherlands; Department of Medical Microbiology, University Medical Center Utrecht, Heidelberglaan 100, 3584 CX Utrecht, The Netherlands; Julius Center for Health Sciences and Primary Care, University Medical Center Utrecht, Heidelberglaan 100, 3584 CX Utrecht, The Netherlands

## Abstract

**Introduction:**

Critically ill patients with sepsis are prone to develop cardiac dysrhythmias, most commonly atrial fibrillation (AF). Systemic inflammation, circulating stress hormones, autonomic dysfunction, and volume shifts are all possible triggers for AF in this setting. We conducted a systematic review to describe the incidence, risk factors and outcomes of new-onset AF in patients with sepsis.

**Methods:**

MEDLINE, EMBASE and Web Of Science were searched for studies reporting the incidence of new-onset AF, atrial flutter or supraventricular tachycardia in patients with sepsis admitted to an intensive care unit, excluding studies that primarily focused on postcardiotomy patients. Studies were assessed for methodological quality using the GRADE system. Risk factors were considered to have a high level of evidence if they were reported in ≥2 studies using multivariable analyses at a *P* value <0.05. Subsequently, the strength of association was classified as strong, moderate or weak, based on the reported odds ratios.

**Results:**

Eleven studies were included. Overall quality was low to moderate. The weighted mean incidence of new-onset AF was 8% (range 0 to 14%), 10% (4 to 23%) and 23% (6 to 46%) in critically ill patients with sepsis, severe sepsis and septic shock, respectively. Independent risk factors with a high level of evidence included advanced age (weak strength of association), white race (moderate association), presence of a respiratory tract infection (weak association), organ failure (moderate association), and pulmonary artery catheter use (moderate association). Protective factors were a history of diabetes mellitus (weak association) and the presence of a urinary tract infection (weak association). New-onset AF was associated with increased short-term mortality in five studies (crude relative effect estimates ranging from 1.96 to 3.32; adjusted effects 1.07 to 3.28). Three studies reported a significantly increased length of stay in the ICU (weighted mean difference 9 days, range 5 to 13 days), whereas an increased risk of ischemic stroke was reported in the single study that looked at this outcome.

**Conclusions:**

New-onset AF is a common consequence of sepsis and is independently associated with poor outcome. Early risk stratification of patients may allow for pharmacological interventions to prevent this complication.

**Electronic supplementary material:**

The online version of this article (doi:10.1186/s13054-014-0688-5) contains supplementary material, which is available to authorized users.

## Introduction

New-onset atrial fibrillation (AF) is a common complication of critical illness, with a reported incidence that varies from 4 to 9% in general intensive care unit (ICU) patients to 32 to 50% in postcardiotomy patients [1-3]. Furthermore, new-onset AF predicts mortality in patients who are hospitalized for heart failure as well as various other critical conditions, although it is possible that AF in these cases is primarily a marker of disease severity rather than a direct cause of death [4-6]. Nonetheless, the development of AF is associated with a sudden reduction in cardiac output and rise in filling pressures and it is, therefore, possible that increased mortality is due to the adverse consequences of AF on cardiac function [4]. In addition, chronic AF is associated with thromboembolic complications, and it is plausible that some of these risks also affect critically ill patients with an acute episode of AF [7].

Although cardiac arrhythmias in the general ICU population have been described since the early 1990s [5,8], most authors have studied unselected cohorts of patients, with neither exclusion of subjects who had a cardiac reason for admission nor those with a known history of chronic or paroxysmal AF. As a consequence, the true incidence and prognosis of new-onset AF in patients presenting with sepsis remains unknown.

Sepsis is characterized by a systemic release of proinflammatory cytokines, high levels of circulating stress hormones, autonomic dysfunction, and may be complicated by organ dysfunction [9,10]. In addition, intravascular volume shifts and cardiovascular compromise will frequently lead to hypotension and elevated lactate levels [11]. All of these features of sepsis are possible triggers for the development of AF [12].

If AF causes poor outcome it might be desirable to start antiarrhythmic prophylaxis in critically ill patients with sepsis in an attempt to prevent this complication. Current guidelines advise the use of beta blockers or amiodarone to prevent postoperative AF in patients following cardiac surgery [13] and it is conceivable that a preemptive strategy could also be effective in patients with (severe) sepsis. Identification of patients at highest risk for AF is therefore important. We aimed to gain better understanding of the incidence, risk factors and outcomes of new-onset AF in critically ill patients with sepsis.

## Methods

### Data sources and search strategy

We searched the literature from 1966 through 2013 using MEDLINE, EMBASE and Web of Science. In our query we used compound search strings for both the determinant (atrial fibrillation) and domain (sepsis) (see Additional file 1 of the online supplemental digital content for full specification of our search query). Only articles published in English, Dutch, French or German were considered for this review. We screened titles and abstracts of identified articles and included all studies describing the incidence, risk factors or outcome of new-onset AF, atrial flutter or other supraventricular tachycardia occurring during ICU stay in adult patients with sepsis, severe sepsis or septic shock. Patients with a supraventricular tachycardia that was not further specified or of unknown origin were included because we expected a high percentage of AF among these patients. Furthermore, we also included reports that primarily focused on patients with severe sepsis and septic shock but that were not specifically restricted to the ICU setting, because we expected a high proportion of critically ill patients to be represented in these studies. We excluded reports that primarily included patients following cardiotomy, that contained no original data, that were published only in abstract form, or that provided no clear definition of the patients or the arrhythmia being studied. Institutional Review Board approval was not sought since our study did not involve human research.

### Quality assessment

Studies that met the inclusion criteria were evaluated for their methodological quality using the Grading of Recommendations Assessment, Development and Evaluation (GRADE) guidelines [14]. The following items were assessed: study design, sample size, domain definition, determinant parameterization, risk factor parameterization and outcome parameterization. We scored all items on a four-point scale from low to high.

### Data analysis

Abstracts were screened for inclusion by the primary author. Articles that were selected for full-text review were assessed by both the first and second author. In case of uncertainty about study inclusion consensus was sought with the third author. Subsequently, data were extracted by the primary author. Where possible, the cumulative incidence of new-onset AF during the sepsis episode, the odds ratios (ORs) for the relations between risk factors and the occurrence of AF, and the OR for the relation between AF and mortality were calculated based on the crude data provided in the article. We calculated weighted mean incidences across studies by summing the reported frequencies of AF in all eligible studies and dividing this by the total number of participants. Risk factors were considered to have a high level of evidence if a significant association (*P* <0.05) was reported in ≥2 studies using multivariable analyses; a moderate level of evidence if a significant association was reported in a single study using a multivariable analysis, or in ≥2 studies using univariable analyses; and a low level of evidence if a significant association was reported in a single study using a univariable analysis. The strength of association was then classified as strong (OR <0.4 or >3.0), moderate (OR 0.4 to 0.7 or 1.5 to 3.0) and weak (OR = 0.7 to 0.9 or OR = 1.1 to 1.5) [15].

## Results

**Figure 1:**
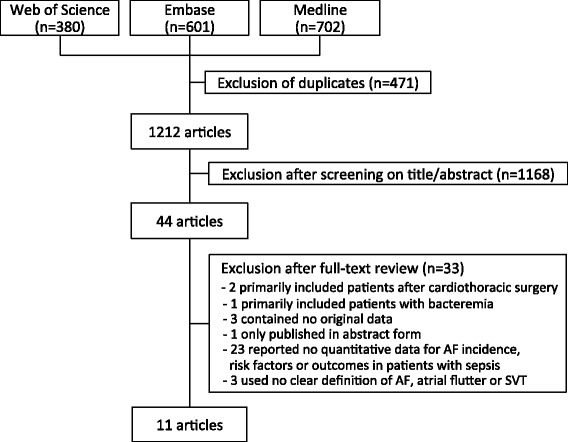
**Flowchart of study selection process.**

Based on our initial search results, 1,212 articles were screened, of which 1,168 were rejected after review of title and abstract. An additional 33 articles were excluded after full review, leaving 11 papers for inclusion (Figure 1) [16-26].

Table 1 shows general characteristics of the included studies. Only six studies used a method of prospective data collection, whereas the others were retrospective analyses of existing databases. The total number of included patients with sepsis varied widely, ranging from 18 to 49,082 per study. The overall methodological quality of the studies was only low to moderate (GRADE scores 2 and 3). See Additional file 2 of the online supplemental digital content for a detailed appraisal of the individual studies. This was mostly due to a retrospective study design, a small sample size, a poor definition of the study domain, an uncertain diagnosis of the determinant (that is, the occurrence of AF), and a lack of multivariable analyses when assessing risk factors. Two studies [24,25] did not explicitly distinguish between patients managed in an ICU or at the ward, yet remained in our review because the provided data suggested that the vast majority of included patients were indeed critically ill.

**Table 1:** **Overview of included studies**

**Reference**	**Setting**	**Number of patients with sepsis/total** ^**a**^	**Detection method AF**	**Outcome**	**Methodological quality** ^**b**^
Arora *et al*., 2007 [16]	Single center, mixed ICU, Australia.	18/61	Prospective bedside detection +12-lead confirmation	n/a	Moderate
Christian *et al*., 2008 [17]	Single center, mixed ICU, USA.	274/274	Retrospective review of monitor data from a database developed for benchmarking	Death	Moderate
Gomez *et al*., 2012 [18]	Multicenter, mixed ICUs, Columbia.	100/100	Prospective detection using telemetry	n/a	Low
Goodman *et al*., 2007 [19]	Single center, mixed ICU, Israel.	149/611	Prospective bedside detection +12-lead confirmation.	n/a	Moderate
Meierhenrich *et al*., 2010 [20]	Single center, surgical ICU, Germany.	50/629	Prospective bedside detection +12-lead confirmation	Death	Moderate
Salman *et al*., 2008 [21]	Single center, mixed ICU, USA.	81/81	Retrospective review of monitor data from a hospital record database	Death	Moderate
Seguin *et al*., 2006 [22]	Single center, trauma ICU, France.	36/293	Prospective bedside detection +12-lead confirmation	n/a	Low
Seguin *et al*., 2004 [23]	Single center, surgical ICU, France.	107/460	Prospective bedside detection +12-lead confirmation	n/a	Moderate
Walkey *et al*., 2011 [24]	Multicenter, acute care hospital, USA.	49,082/49,082	Retrospective review from an administrative claims database using ICD-9-CM codes for detection of AF	Death	Moderate
				Stroke	
Walkey *et al*., 2013 [25]^c^	Multicenter, Medicare hospital, USA.	40,740/40,740^d^	Retrospective review from an administrative claims database using ICD-9-CM codes for detection of AF	n/a	Moderate
Wells *et al*., 2011 [26]	Single center, medical ICU, USA.	465/1466	Retrospective review from an administrative database, detection of AF using telemetry	Death	Low

^a^When studies differentiate between sepsis, severe sepsis and septic shock, the number of patients with sepsis equals the number of all patients with sepsis including patients with severe sepsis and septic shock; ^b^see Additional file 2 for a detailed appraisal of the individual studies; ^c^Walkey 2013 [25] only excluded patients after cardiothoracic surgery in a sensitivity analysis for identification of the risk factors, therefore we used this study only for the analysis of risk factors; ^d^the number of patients with sepsis excluding patients with cardiothoracic surgery or endocarditis during the sepsis hospitalization. AF: atrial fibrillation; ICD-9-CM: International Classification of Diseases-Ninth Revision-Clinical Modification; ICU: intensive care unit; n/a: not available.

Table 2 shows the incidence of new-onset AF in patients with sepsis by various stages of disease progression. The weighted mean incidence of new-onset AF was 8% (range 0 to 14%), 10% (4 to 23%) and 23% (6 to 46%) in patients with sepsis, severe sepsis and septic shock, respectively. Not all studies reported the incidences of new-onset AF for the various disease stages separately. Three studies reported only a combined incidence for patients with either sepsis or severe sepsis (mean 5%, range 3 to 10%) [17,22,23]; one study reported an incidence for patients with either severe sepsis or septic shock (28%) [26]; and another two studies reported an incidence for patients with sepsis of unspecified severity (mean 28%; range 25 to 50%) [16,19]. In contrast, a single large study that was not formally restricted to the ICU setting only, and that used International Classification of Diseases-Ninth Revision-Clinical Modification (ICD-9-CM) codes to detect episodes of new-onset AF, reported an incidence of 5% in hospitalized patients with severe sepsis and 6% in patients with septic shock. No studies reported the number of recurrent AF episodes per ICU admission, their duration, or their hemodynamic consequences. Two studies reported the therapeutic interventions that were performed in response to AF onset in patients with sepsis, including electrical cardioversion (31%) and the initiation of antiarrhythmic drugs such as amiodarone (73%), digoxin (63%), bêta blockers (51%), and other/unspecified medications for rate-control (52%) or pharmacologically cardioversion (12%) [20,21].

**Table 2:** **Incidence of new-onset atrial fibrillation in patients with various stages of sepsis**

**Reference**	**Disease stage**		
	**Sepsis**	**Severe sepsis**	**Septic shock**
**ICU population**			
Arora *et al*., 2007 [16]	[ - - - - - - - - - - - - - - - - - - - - - - - - - - - - - - - - - - - - - - - - - - 9/18 (50%) - - - - - - - - - - - - - - - - - - - - - - - - - - - - - - - - - - - - - - - - - - -]		
Christian *et al*., 2008 [17]	6/184 (3%)		10/90 (11%)
Gomez *et a*l., 2012 [18]	0/10 (0%)	1/28 (4%)	4/62 (6%)
Goodman *et al*., 2007 [19]	[ - - - - - - - - - - - - - - - - - - - - - - - - - - - - - - - - - - - - - - - - - 37/149 (25%) - - - - - - - - - - - - - - - - - - - - - - - - - - - - - - - - - - - - - - - - - -]		
Meierhenrich *et al*., 2010 [20]			23/50 (46%)
Salman *et al*., 2008 [21]	2/14 (14%)	3/13 (23%)	20/54 (37%)
Seguin *et al*., 2006 [22]	3/29 (10%)		2/7 (29%)
Seguin *et al*., 2004 [23]	5/84 (6%)		7/23 (30%)
Wells *et al*., 2011 [26]		132/465 (28%)	
**Mixed population**			
Walkey *et al*., 2011 [24]*		1074/20253 (5%)	1822/28829 (6%)

The numerators of the fractions in this table display the number of patients with new-onset AF, the denominators of the fractions display the total number of patients at risk in the specific sepsis stages. Whenever the incidence of new-onset AF for the various stages of sepsis was not reported separately, the combined incidences are shown. *This study included hospitalized patients with severe sepsis and septic shock, based on ICD-9-CM discharge codes; although most patients had been admitted to the ICU, the study was not formally restricted to critically ill patients only (source: personal communication by the author). AF: atrial fibrillation; ICD-9-CM: International Classification of Diseases-Ninth Revision-Clinical Modification; ICU: intensive care unit.

Table 3 shows the risk factors for the development of new-onset AF in patients with sepsis that were reported at least once at a significance level <0.05 (see Additional file 3 of the online data supplement for a complete overview of all potential risk factors). Risk factors with a high level of evidence included advanced age, white race, presence of a respiratory tract infection, organ failure, and use of a pulmonary artery catheter. In contrast, the incidence of AF was reduced in patients with diabetes mellitus and those with urinary tract infections. Conflicting results were found for a history of hypertension and chronic obstructive pulmonary disease (COPD).

**Table 3:** **Level of evidence and strength of association of selected risk factors for new-onset atrial fibrillation in patients with sepsis**

**Risk factor category**	**Variables**	**Level of evidence** ^**a**^	**Strength of association** ^**b**^							
			**Univariable analysis**					**Multivariable analysis**		
			**Strength**				**References** ^**d**^	**Strength**		**References** ^**d**^
Demographics	Increased age, per 10 years	●●●	n/a				[17,20,21,24,26]	M +	W +	[24,25]
	Male gender	●●○	M +	M +	M +	W +	[20,21,24,26]	W +	No	[24,25]
	White race	●●●	S +	M +	M +		[21,24,26]	M +	M +	[24,25]
Comorbidities	COPD	●●○	S +	M +	W +	W +	[20,21,24,26]	W -		[25]
	Diabetes mellitus	●●●	M -	W -	W +		[21,24,26]	W -	W -	[24,25]
	Obesity	●●○	W -				[24]	W +		[24]
	Heart failure	●●○	M +	M +			[20,24]	M +	No	[24,25]
	Hypertension	●○○	S +	W +	W -		[20,21,24]	W -	No	[24,25]
	Coronary artery disease	●○○	S +	M +	M +		[20,21,26]			
	Myocardial infarction	●○○	W +				[24]			
	Stroke	●●○	M +	M +			[21,24]	M +		[24]
	Renal disease	●●○						W -		[25]
	Malignancy	●●○	W +				[24]	W +		[25]
Source of infection	Primary blood stream	●●○	W -				[24]	W +		[24]
	Respiratory tract	●●●	M +				[24]	W +	W +	[24,25]
	Abdominal	●●○	M +				[24]	M +		[24]
	Urinary tract	●●●	W -				[24]	W -	W -	[24,25]
	Skin or soft tissue	●●○	No				[24]	W +	W -	[24,25]
Pathogen	Fungal	●●○	M +				[24]	M +		[24]
	Gram-positive bacteria	●●○	W +				[24]	W +		[24]
Severity of disease	Organ failure^c^	●●●	S +	M +	n/a	n/a	[17,24,20,21]	M +	M +	[24,25]
	Electrolyte abnormality	●○○	W +				[24]			
Critical care interventions	Pulmonary artery catheter use	●●●	S +	S +			[17,24]	M +	W +	[24,25]
	Mechanical ventilation	●●○	S +				[17]	W +		[24]

^a^Level of evidence: high (●●●): risk factor reported in ≥2 references using multivariable analyses at a *P* value <0.05; Moderate (●●○): risk factor reported in 1 reference using multivariable analysis at a *P* value <0.05 or ≥2 references using univariable analyses at a *P* value <0.05; low (●○○): risk factor reported in 1 reference using univariable analysis at a *P* value <0.05; ^b^strength of association: Strong positive association (S+): OR >3; Moderate positive association (M+): OR = 1.5 to 3.0; Weak positive association (W+): OR <1.5; Strong negative association (S-): OR <0.4; Moderate negative association (M-): OR = 0.4 to 0.7; Weak negative association (W-): OR >0.7; No association (No): OR = 0.9-1.1 (based on [15]); ^c^this includes high APACHE II, APS or SOFA scores, or the presence of shock upon ICU admission; ^d^the references refer to the columns showing the strength of association. APACHE II: Acute Physiological and Chronic Health Evaluation II; APS: Acute Physiology Score; COPD: chronic obstructive pulmonary disease; ICU: intensive care unit; n/a: not available; OR: odds ratio; SOFA: Sequential Organ Failure Assessment.

Table 4 shows outcomes for patients with at least a single episode of new-onset AF as compared to patients with maintained sinus rhythm. All studies reporting mortality found increased case fatality rates in patients who had developed AF during their stay. Estimated ORs varied from 1.96 (95% confidence interval (CI) 1.26 to 3.03) to 3.32 (95% CI 1.12 to 9.84) for acute (that is, ICU or in-hospital) mortality, and from 2.25 (95% CI 0.66 to 7.73) to 4.29 (95% CI 1.53 to 11.97) for 28-day mortality. Only two studies reported a measure of association that was corrected for baseline imbalances in severity of illness, with an adjusted relative risk (RR) 1.07 (95% CI 1.04 to 1.11) for in-hospital mortality and adjusted OR 3.28 (95% CI 1.13 to 9.57) for 28-day mortality, respectively [21,24]. Three studies reported a significantly increased length of stay for patients who had experienced an episode of new-onset AF in the ICU (weighted mean difference 9 days, reported range 5 to 13 days) [17,20,21]. An increased risk for in-hospital ischemic stroke was found in a single study that reported this outcome (adjusted OR 2.70, 95% CI 2.05 to 3.57) [24].

**Table 4:** **Outcomes of new-onset atrial fibrillation in patients with sepsis, severe sepsis or septic shock**

**Reference**	**Outcome**	**Patients with new-onset AF** **(number of patients with outcome/total)**	**Patients without new-onset AF** **(number of patients with outcome/total)**	**Crude OR**	***P*** **value**	**Adjusted OR**	***P*** **value**
Christian *et al.,* 2008 [17]	ICU mortality	11/16 (69%)	102/256 (40%)	3.32 (1.12- 9.84)	0.03		
Meierhenrich *et al*., 2010 [20]	ICU mortality	10/23 (44%)	6/27 (22%)	2.69 (0.79-9.17)	0.14		
	28-day mortality	9/23 (39%)	6/27 (22%)	2.25 (0.66-7.73)	0.22		
Salman *et al*., 2008 [21]	ICU mortality	12/25 (48%)	15/56 (27%)	2.52 (0.95-6.74)	0.06		
	In-hospital mortality	16/25 (65%)	20/56 (38%)	3.20 (1.20-8.55)	0.02		
	28-day mortality	18/25 (72%)	21/56 (38%)	4.29 (1.53-11.97)	0.004	3.28 (1.13-9.57)^a^	0.03
Walkey *et al*., 2011 [24]	In-hospital mortality	1629/2896 (56%)	13652/36200 (38%)	2.12 (1.97-2.29)	<.0001	1.07 (1.04-1.11)^b,c^	n/a
	In-hospital ischemic stroke	75/2896 (3%)	249/36200 (1%)	3.84 (2.96-4.98)	<.0001	2.70 (2.05-3.57)^d^	n/a
Wells *et al*., 2011 [26]	ICU/in-hospital mortality	95/132 (72%)	189/333 (57%)	1.96 (1.26-3.03)	0.002		

Data are expressed as absolute numbers (%) unless other specified. ^a^Adjusted for the severity of illness at ICU admission measure by the Acute Physiology and Chronic Health Evaluation (APACHE) III predicted mortality rate; ^b^adjusted for: age, sex, race/ethnicity, history of diabetes mellitus, hypertension, obesity, heart failure, stroke, myocardial infarction, chronic obstructive pulmonary disease, metastatic or hematologic malignancy, number of organ failures, presence of electrolyte disturbance, source of sepsis, type of organ failure, type of pathogenic organism and use of pulmonary artery catheter; ^c^relative risk; ^d^adjusted for: age, sex, race/ethnicity, history of diabetes mellitus, hypertension, obesity, heart failure, stroke, myocardial infarction, chronic obstructive pulmonary disease, metastatic or hematologic malignancy, number of organ failures, presence of electrolyte disturbance, source of sepsis, type of organ failure, type of pathogenic organism and use of pulmonary artery catheter. AF: atrial fibrillation; ICU: intensive care unit; n/a: not available; OR: odds ratio.

## Discussion

The reported incidence of new-onset AF varies greatly in critically ill patients with sepsis, but consistently rises with progression from milder stages of the disease to shock. Furthermore, the occurrence of even a single episode of AF is associated with increased mortality, increased length of stay and - possibly - an increased risk of stroke in these patients, both in crude and adjusted analyses. This suggests that new-onset AF is causally linked to poor outcome in critically ill patients with sepsis.

The incidences of new-onset AF in patients with sepsis admitted to the ICU that we found in our systematic review are higher than the 4 to 9% occurrence rates reported for general ICU populations, but lower than the estimated 30 to 50% rate in postcardiotomy patients [1-3]. Furthermore, the reported incidences in our review varied widely between studies, which may be explained by random variation due to the small sample sizes of most included studies, but also by differences in patient populations and by misclassification (that is, not all episodes of AF may have been correctly detected). In fact, the domain was not clearly defined in five studies, and some did not explicitly exclude patients who developed sepsis following cardiac surgery [18,22-24,26]. In addition, another seven studies did not explicitly exclude patients with a prior history of chronic or paroxysmal AF [16,18,21-24,26]. Both of these shortcomings may have led to an overestimation of the AF incidence due to sepsis. Of note, a single large study did not explicitly stipulate ICU admission as an entry requirement, yet was included in our analyses of risk factors and outcomes since most patients with severe sepsis or septic shock were expected to be critically ill indeed [24]. However, given the uncertainty about domain, this study was excluded from the calculation of pooled incidences of new-onset AF in an ICU setting.

Misclassifications might have also occurred due to differences in the methods used for diagnosing AF. Prospective use of continuous bedside monitoring can detect even short episodes of new-onset AF that may have only limited clinical relevance, whereas retrospective classification of AF based on available administrative databases will likely result in underreporting. Indeed, the three retrospective studies found lower incidences than most of the prospective studies in our review [17,21,24].

Risk factors for the development of AF in patients with sepsis were reported in only six of the included studies [17,20,21,24-26]. Markers of illness severity (such as the presence of organ failures and shock) as well as several critical care interventions were associated with an increased risk of AF. These findings give support for the general notion that AF may be triggered by high levels of circulating proinflammatory cytokines, catecholaminergic stress electrolyte imbalances, and a disrupted volume status during sepsis. In addition, known risk factors for chronic or paroxysmal AF in the general population, such as advanced age, white race, male gender, obesity and (ischemic) heart failure, were also associated with the development of AF during sepsis [27,28]. However, in contrast to reported associations in the general population, the included studies did not identify hypertension, valvular heart disease or diabetes as significant risk factors [27-29]. In fact, two studies paradoxically reported that diabetes mellitus was associated with a decreased risk of AF onset [24,25]. It might be that patients with diabetes mellitus who did not develop paroxysmal or chronic AF prior to their acute illness were characterized by some (unknown) factors that protected them from developing this dysrhythmia during the sepsis event. Conflicting results were found for a history of hypertension and COPD [20,24-26]. The authors reporting an (unexpected) reduced risk of AF stated that this finding may have been an artifact that was due to underreporting of chronic comorbidities in their data [24,25]. However, in that case we would not expect a protective effect.

Outcomes of new-onset AF in patients with sepsis were described in five studies [17,20,21,24,26]. All studies consistently reported worse outcome in patients with new-onset AF as compared to patients with preserved sinus rhythm, although only two studies used multivariate analyses to adjust for baseline imbalances [21,24]. Even then, residual confounding in both studies is likely since only limited numbers of covariables were collected and time-dependent variations in the evolution of disease severity prior to the onset of AF were ignored. Methodologically advanced analytical approaches are necessary to demonstrate genuinely independent associations with morbidity and mortality. In the end, therefore, it still remains somewhat elusive to what extent new-onset AF in patients with sepsis truly impacts outcome. However, given the consistency of the findings across settings and biological plausibility, a relation that is (at least in part) causal seems likely.

Additional morbidity and mortality following the development of AF in patients with sepsis may be explained by the decrease in cardiac output and blood pressure that occurs in most patients due to reduced left ventricular filling. This is particularly the case in the presence of rapid ventricular response rates. The resulting hemodynamic compromise may impair the recovery of organ function in patients with severe sepsis or shock. Furthermore, AF has been associated with the development of intracardiac thrombi, posing a subsequent risk of systemic embolization and stroke [30]. Harm, however, may also result from the indiscriminate use of anticoagulants in patients with AF, particularly in an ICU setting. Unfortunately, there are presently no evidence-based guidelines for the use of anticoagulant prophylaxis in these patients [10,31].

Taken together, the management of new-onset AF in critically ill patients with sepsis poses a major clinical challenge. It may therefore be desirable to start pharmacological prophylaxis in an attempt to prevent this complication rather than to be reactive, provided that it is possible to adequately identify patients who are at highest risk for developing AF during sepsis. A recent study investigating the effect of esmolol in patients with septic shock gives support to the feasibility of such approach [32]. Although reducing heart rate is likely to improve cardiovascular function, treating sinus tachycardia - and thereby possibly preventing AF - in patients with sepsis remains controversial, however, and further research is necessary before any recommendations regarding pharmacological prophylaxis can be made.

Our review is the first to give a systematic overview of the reported incidences, risk factors and outcomes associated with AF during sepsis in the ICU. However, our review has several limitations, primarily because the majority of the included papers pertained to single center studies with small sample sizes, whereas the few multicenter studies that were included had retrospective designs and used only administrative data (such as ICD-9-CM codes) for the classification of both sepsis and new-onset AF. In addition, many studies did not discriminate between the various sepsis stages when reporting the occurrence of new-onset AF [16,17,19,22,23,26]. Furthermore, there was large statistical heterogeneity as well as variability in clinical settings amongst studies. This precluded pooling of data into a meta-analysis. At last, it still remains uncertain whether new-onset AF in patients with sepsis is merely a marker for severity of disease, or whether it truly impacts outcome. Therefore further research is warranted to demonstrate independent associations with morbidity and mortality.

## Conclusions

New-onset AF is a common occurrence in critically ill patients with sepsis, and its incidence rises with increasing severity of disease. Multivariable analyses suggest that it is independently associated with poor outcome, but whether this relation is truly causal remains difficult to establish. In view of these findings there is a need for better quality observational studies, because reliable identification of patients with sepsis who are prone for the development of AF may allow for early pharmacological interventions to prevent this complication.

## Key messages

- New-onset AF is a common complication in critically ill patients with sepsis and its incidence increases with progression of the disease stage.
- New-onset AF is independently associated with a risk of stroke, a prolonged length of stay in the ICU, and increased mortality.
- Early identification of patients who are at increased risk for developing AF seems feasible and may allow for pharmacological interventions to prevent this complication.

## Additional files

Additional file 1: eTable 1. Search query. Additional file 2: eTable 2. Methodological quality of selected studies. Additional file 3: eTable 3. Risk factors for new-onset atrial fibrillation in patients with sepsis, severe sepsis or septic shock.

## Abbreviations

AF: atrial fibrillation
CI: confidence interval
COPD: chronic obstructive pulmonary disease
GRADE: Grading of Recommendations Assessment, Development and Evaluation
ICD-9-CM: International Classification of Diseases-Ninth Revision-Clinical Modification
ICU: intensive care unit
OR: odds ratio
RR: relative risk
