# Supplementary material for: Incidence, risk factors and outcomes of new-onset atrial fibrillation in patients with sepsis: a systematic review
Source: Crit Care. 2014 Dec 15;18(6):688. doi: 10.1186/s13054-014-0688-5 (PMC4296551; doi:10.1186/s13054-014-0688-5)
Supplement: Additional file 1: eTable 1. — Search query. [file 13054_2014_688_MOESM1_ESM.pdf]

eTable 1. Search query

MEDLINE	Search terms	n
#1 Determinant: Atrial fibrillation	'atrial fibrillation' [MeSH] OR 'cardiac arrhythmias' [MeSH] OR 'supraventricular tachycardia' [MeSH] OR 'atrial fibrillation' [Title/Abstract] OR 'auricular fibrillation' [Title/Abstract] OR 'cardiac arrhythmia' [Title/Abstract] OR 'cardiac dysrhythmia' [Title/Abstract] OR 'supraventricular tachycardia' [Title/Abstract] OR 'supraventricular arrhythmia' [Title/Abstract]	
#2 Domain: Sepsis	'sepsis' [MeSH]' OR 'septic shock' [MeSH] OR 'sepsis' [Title/Abstract] OR 'septicemia' [Title/Abstract] OR 'septicaemia' [Title/Abstract] OR 'severe sepsis' [Title/Abstract] OR 'septic shock' [Title/Abstract]	
Combined set	#1 AND #2	702

MeSH: Medical Subject Headings.

EMBASE	Search terms	n
#1 Determinant: Atrial fibrillation	'atrial fibrillation':ab,ti OR 'auricular fibrillation':ab,ti OR 'cardiac arrhythmia':ab,ti OR 'cardiac dysrhythmia':ab,ti OR 'supraventricular tachycardia':ab,ti OR 'supraventricular arrhythmia':ab,ti	
#2 Domain: Sepsis	'sepsis':ab,ti OR 'septicemia':ab,ti OR 'septicaemia':ab,ti OR 'severe sepsis':ab,ti OR 'septic shock':ab,ti	
Combined set	#1 AND #2	601

Web of Science	Search terms	n
#1 Determinant: Atrial fibrillation	Topic=(Atrial Fibrillation) OR Topic=(Auricular Fibrillation) OR Topic=(Cardiac Arrhythmia) OR Topic=(Cardiac Dysrhythmia) OR Topic=(Supraventricular Tachycardia) OR Topic=(Supraventricular arrhythmia)	
#2 Domain: Sepsis	Topic=(Sepsis) OR Topic=(Septicemia) OR Topic=(Septicaemia) OR Topic=(Severe Sepsis) OR Topic=(Septic Shock)	
Combined set	#1 AND #2	380
