# Supplementary material for: Incidence, risk factors and outcomes of new-onset atrial fibrillation in patients with sepsis: a systematic review
Source: Crit Care. 2014 Dec 15;18(6):688. doi: 10.1186/s13054-014-0688-5 (PMC4296551; doi:10.1186/s13054-014-0688-5)
Supplement: Additional file 2: eTable 2. — Methodological quality of selected studies. [file 13054_2014_688_MOESM2_ESM.pdf]

eTable 2. Methodological quality of selected studies

Reference	Study design	Sample size (patients with sepsis)	Domain definition	Determinant parameterization	Risk factor parameterization	Outcome parameterization	Overall quality score[a]
**Arora 2007**	Low-moderate (2.5)<br>– Observational (-2)<br>– Prospective (+0.5)	Very low (1)<br>– n=18 (-3)	Moderate (3)<br>– No distinction between sepsis, severe sepsis and septic shock (-0.5)<br>– No explicit exclusion of patients with a prior history of AF (-0.5)	Moderate-high (3.5)<br>– Duration of AF not reported (-0.5)	(n/a)	(n/a)	Moderate (2.5)
**Christian 2008**	Low (2)<br>– Observational (-2)	Low-moderate (2.5)<br>– n=274 (-1.5)	Moderate (3)<br>– No description of sepsis diagnosis (-1)	Low-moderate (2.5)<br>– Retrospective diagnosis of AF (-1)<br>– Duration of AF not reported (-0.5)	Moderate (3)<br>– Univariable analysis (-1)	Moderate (3)<br>– Univariable analysis (-1)	Moderate (2.7)
**Gomez 2012**	Low-moderate (2.5)<br>– Observational (-2)<br>– Prospective (+0.5)	Low (2)<br>– n= 100 (-2)	Very low (1)<br>– No description of sepsis diagnosis (-1)<br>– No exclusion of postcardiotomy patients (-1)<br>– No exclusion of patients with a prior history of AF (-1)	Moderate-high (3.5)<br>– Duration of AF not reported (-0.5)	(n/a)	(n/a)	Low (2.3)
**Goodman 2007**	Low-moderate (2.5)<br>– Observational (-2)<br>– Prospective (+0.5)	Low (2)<br>– n=149 (-2)	Low-moderate (2.5)<br>– No description of sepsis diagnosis (-1)<br>– No distinction between SIRS and sepsis (-0.5)	High (4)	(n/a)	(n/a)	Moderate (2.8)
**Meierhenrich 2010**	Low-moderate (2.5)<br>– Observational (-2)<br>– Prospective (+0.5)	Very low-low (1.5)<br>– n=50 (-2.5)	High (4)	Moderate-high (3.5)<br>– Duration of AF not reported (-0.5)	Moderate (3)<br>– Univariable analysis (-1)	Moderate (3)<br>– Univariable analysis (-1)	Moderate (2.9)
**Salman 2008**	Low (2)<br>– Observational (-2)	Low (2)<br>– n=81 (-2)	Moderate (3)<br>– No exclusion of patients with a prior history of AF (-1)	Low-moderate (2.5)<br>– Retrospective diagnosis of AF (-1)<br>– Duration of AF not reported (-0.5)	Moderate (3)<br>– Univariable analysis (-1)	Moderate (3)<br>– Univariable analysis (-1)	Moderate (2.6)
**Seguin 2006**	Low-moderate (2.5)<br>– Observational (-2)<br>– Prospective (+0.5)	Very low (1)<br>– n=36 (-3)	Very low-low (1.5)<br>– Only trauma patients (-1)<br>– No exclusion of postcardiotomy patients (-1)<br>– No explicit exclusion of patients with a prior history of AF (-0.5)	High (4)	(n/a)	(n/a)	Low (2.3)
**Seguin 2004**	Low-moderate (2.5)<br>– Observational (-2)<br>– Prospective (+0.5)	Low (2)<br>– n= 107 (-2)	Low-moderate (2.5)<br>– No exclusion of postcardiotomy patients (-1)<br>– No explicit exclusion of patients with a prior history of AF (-0.5)	Moderate-high (3.5)<br>– Duration of AF not reported (-0.5)	(n/a)	(n/a)	Moderate (2.6)
**Walkey 2011**	Low (2)<br>– Observational (-2)	High (4)<br>– n=49082 (-0)	Very low-low (1.5)<br>– Use of ICD-9-codes to diagnose sepsis (-1)<br>– No exclusion of postcardiotomy patients (-1)<br>– No explicit exclusion of patients with a prior history of AF (-0.5)	Low-moderate (2.5)<br>– Use of ICD-9-codes to diagnose AF (-1)<br>– Duration of AF not reported (-0.5)	High (4)	High (4)	Moderate (3)
**Walkey 2013**	Low (2)<br>– Observational (-2)	High (4)<br>– n=60209 (-0)	Low (2)<br>– Use of ICD-9-codes to diagnose sepsis (-1)<br>– Only exclusion of postcardiotomy patients in sensitivity analysis (-1)	Low-moderate (2.5)<br>– Use of ICD-9-codes to diagnose AF (-1)<br>– Duration of AF not reported (-0.5)	High (4)	(n/a)	Moderate (2.9)
**Wells 2011**	Low (2)<br>– Observational (-2)	Moderate (3)<br>– n=465 (-1)	Very low (1)<br>– Use of ICD-9-codes to diagnose sepsis (-1)<br>– No exclusion of postcardiotomy patients (-1)<br>– No exclusion of patients with a prior history of AF (-1)	Low-moderate (2.5)<br>– Retrospective diagnosis of AF (-1)<br>– Duration of AF not reported (-0.5)	Moderate (3)<br>– Univariable analysis (-1)	Moderate (3)<br>– Univariable analysis (-1)	Low (2.4)

AF: atrial fibrillation; ICD-9-CM: international classification of diseases-ninth revision-clinical modification; n/a: not available.

[a] Studies were evaluated for quality using the GRADE guidelines (Balshem, 2011). The following items were appraised: study design, sample size, domain definition and determinant parameterization. These items were scored on a scale from a score form our (high) to one point (low). We used a maximal score of 4 points, for every weak point of the study we subtracted points and for every strong point of the study we gave bonus points. With the sum of the scored items we calculated a mean overall quality score of the concerning study.
