# Supplementary material for: Incidence, risk factors and outcomes of new-onset atrial fibrillation in patients with sepsis: a systematic review
Source: Crit Care. 2014 Dec 15;18(6):688. doi: 10.1186/s13054-014-0688-5 (PMC4296551; doi:10.1186/s13054-014-0688-5)
Supplement: Additional file 3: eTable 3. — Risk factors for new-onset atrial fibrillation in patients with sepsis, severe sepsis or septic shock. [file 13054_2014_688_MOESM3_ESM.pdf]

eTable 3. Risk factors for new-onset atrial fibrillation in patients with sepsis

Risk factor	Patients with new-onset AF	Patients without new-onset AF	Crude OR (95% CI)	p-value	Adjusted OR (95% CI)	p-value
**Christian 2008**						
Number at risk	16	256				
Age, y	65.6 ± 12.3	62.2 ± 16.5		0.4		
Surgery (≤7 days before admission)	7 (44)	82 (32)	1.65 (0.59-4.59)	0.41		
Severity of illness						
APACHE II	0.49 ± 0.17	0.55 ± 0.26		0.36		
Shock	10 (63)	80 (31)	3.67 (1.29-10.44)	0.01		
Critical care interventions						
Mechanical ventilation	16 (100)	154 (60)	21.19 (1.26-357.68)	<0.01		
Pulmonary artery catheter	11 (69)	90 (35)	4.06 (1.37-12.04)	0.01		
**Meierhenrich 2010**						
Number at risk	23	27				
Age, y	66 (41-85)	56 (18-80)		<0.01		
Gender (female)	5 (22)	12 (44)	0.35 (0.10-1.21)	0.14		
Comorbidities						
COPD	4 (17)	1 (4)	5.47 (0.57-52.97)	0.17		
Coronary artery disease	5 (22)	2 (7)	3.47 (0.60-19.95)	0.23		
Heart failure	1 (4)	0 (0)	2.45 (0.08-76.64)	0.46		
Hypertension	17 (74)	11 (41)	4.12 (1.23-13.77)	0.02		
Valvular disease	1 (4)	1 (4)	1.18 (0.07-20.01)	1.00		
Chronic drug use						
ACE inhibitor	2 (9)	7 (26)	0.27 (0.05-1.47)	0.15		
β-blocker	8 (35)	6 (22)	1.87 (0.54-6.51)	0.36		
Calcium antagonist	7 (30)	2 (7)	5.47 (1.01-29.7)	0.06		
Digoxin	1 (4)	0 (0)	2.43 (0.21-28.58)	0.46		
Surgery						
Abdominal	12 (52)	19 (70)	0.46 (0.14-1.47)			
Aortic	4 (17)	1 (4)	4.47 (0.57-52.97)			
Lung	4 (17)	2 (7)	2.63 (0.44-15.91)			
Neuro	1 (4)	1 (4)	1.18 (0.07-20.01)			
Traumatologic	0 (0)	2 (7)	-			
Vascular	0 (0)	2 (7)	-			
Other	2 (9)	0 (0)	-			

Severity of illness					
CRP level, max (mg/dl)	288 (72-483)	273 (23-214)		0.28	
SAPS II	31 (15-63)	30 (12-65)		0.12	
SOFA max	12 (7-17)	9 (5-18)		0.01	
Critical care interventions					
Dobutamine	10 (44)	6 (22)	2.69 (0.79-9.17)	0.14	
Noradrenaline, max (μg/kg/min)	0.50 (0.15-2.00)	0.30 (0.15-1.40)		0.13	
**Salman 2008**					
**Number at risk**	**25**	**56**			
Age, y	67.0 ± 9.6	55.7 ± 15.0		<0.01	
Gender (female)	7 (28)	28 (50)	0.39 (0.14-1.08)	0.06	
White race	25 (100)	52 (93)	3.85 (0.20-75.60)	0.31	
Surgical patients	1 (4)	4 (7)	0.54 (0.06-5.11)	1.00	
Comorbidities					
Cerebrovascular disease	2 (8)	2 (4)	2.35 (0.31-17.70)	0.59	
COPD	1 (4)	1 (2)	2.29 (0.14-38-18)	0.52	
Coronary artery disease	2 (8)	2 (4)	2.35 (0.31-17.70)	0.59	
Diabetes mellitus	4 (16)	15 (27)	0.52 (0.15-1.77)	0.40	
Hypertension	11 (44)	21 (38)	1.31 (0.50-3.41)	0.58	
Previous PAF	4 (16)	1 (2)	10.48(1.11-99.22)	0.03	
Severity of illness					
Acute Physiology Score	85.0 ± 29.6	68.7 ± 32.4		0.03	
APACHE III	106.0 ± 31.9	85.0 ± 33.0		0.01	
**Walkey 2011**					
**Number at risk**	**2,896**	**36,200**			
Age, y	74 ± 12	66 ± 17			1.52 (1.47-1.56)[a]
Gender (female)	1,280 (44)	17,690 (49)	0.83 (0.77-0.89)		0.83 (0.76-0.90)
Race					
White	1,861 (64)	19,006 (53)	1.63 (1.50-1.76)		1 (reference)
Black	209 (7)	3,547 (10)	0.72 (0.62-0.83)		0.67 (0.58-0.78)
Hispanic	384 (13)	8,045 (22)	0.54 (0.48-0.60)		0.58 (0.50-0.63)
Other	442 (15)	5,502 (15)	1.00 (0.90-1.12)		0.78 (0.69-0.87)
Comorbidities					
Congestive heart failure	306 (11)	2,312 (6)	1.73 (1.53-1.96)		1.61 (1.41-1.83)
COPD	189 (7)	1,920 (5)	1.25 (1.07-1.45)		
Diabetes mellitus	800 (28)	12,135 (34)	0.76 (0.70-0.82)		0.82 (0.75-0.90)
Hypertension	1,336 (46)	17,373 (48)	0.93 (0.86-1.00)		0.88 (0.81-0.95)
Malignancy	189 (7)	1,920 (5)	1.25 (1.07-1.45)		1.23 (1.09-1.39)
Myocardial infarction	165 (6)	1,659 (5)	1.26 (1.07-1.48)		
Obesity	208 (7)	2,914 (8)	0.88 (0.76-1.02)		1.20 (1.03-1.40)
Stroke	126 (4)	880 (2)	1.83 (1.51-2.21)		1.64 (1.35-2.01)
Severity of illness					
Total organ failures	3.10 ±1.28	2.44 ±1.35			
Circulatory failure	2,049 (71)	23,264 (64)	1.35 (1.24-1.46)		
Hematologic failure	933 (32)	7,301 (20)	1.88 (1.73-2.04)		1.50 (1.34-1.68)
Hepatic failure	334 (12)	3,150 (9)	1.37 (1.21-1.54)		
Metabolic failure	706 (24)	7,246 (20)	1.29 (1.18-1.41)		
Neurologic failure	526 (18)	5,318 (15)	1.29 (1.17-1.42)		
Renal failure	2,048 (71)	21,343 (59)	1.68 (1.55-1.83)		1.40 (1.26-1.56)
Respiratory failure	2,385 (82)	20,833 (58)	3.44 (3.12-3.80)		2.81 (2.48-3.19)
Electrolyte abnormality	2,051 (71)	23,963 (66)	1.24 (1.14-1.35)		
Critical care interventions					
Pulmonary artery catheter	172 (6)	722 (2)	3.10 (2.62-3.68)		2.25 (1.87-2.70)
Source of infection					
Abdominal	735 (25)	5,532 (15)	1.89 (1.73-2.06)		1.77 (1.59-1.97)
Primary bacteremia	600 (21)	8,910 (25)	0.80 (0.73-0.88)		1.17 (1.02-1.36)
Respiratory tract	1,419 (49)	14,178 (39)	1.49 (1.38-1.61)		1.27 (1.14-1.40)
Skin or soft tissue	229 (8)	2,888 (8)	0.99 (0.86-1.14)		1.33 (1.14-1.55)
Urinary tract	950 (33)	13,748 (38)	0.80 (0.74-0.86)		0.89 (0.81-0.99)

Pathogen type						
Fungal	104 (4)	632 (2)	2.10 (1.70-2.59)		1.59 (1.27-2.00)	
Gram-negative bacteria	677 (23)	8,776 (24)	0.95 (0.87-1.04)			
Gram-positive bacteria	822 (28)	8,675 (24)	1.26 (1.16-1.37)		1.29 (1.18-1.55)	
Not specified	1,538 (53)	20,268 (56)	0.89 (0.83-0.96)			
**Walkey 2013**						
Total number of patients	40,740					
Age,y						
67-74					1.00 (reference)	
75-84					1.34 (1.25-1.45)	<0.01
≥ 85					1.69 (1.57-1.83)	<0.01
Gender (female)					0.99 (0.93-1.04)	0.66
Race						
Black					0.65 (0.59-0.72)	<0.01
White					1.00 (reference)	
Other					0.65 (0.56-0.76)	<0.01
Comorbidities						
COPD					0.92 (0.86-0.98)	0.01
Diabetes mellitus					0.92 (0.87-0.98)	0.01
Heart failure					0.97 (0.91-1.04)	0.38
Hypertension					0.97 (0.90-1.04)	0.36
Renal disease					0.81 (0.75-0.88)	<0.01
Valvular heart disease					0.98 (0.91-1.06)	0.65
Severity of illness						
Any organ failure					1.26 (1.17-1.36)	<0.01
ICU admission					2.04 (1.90-2.19)	<0.01
New-onset dialysis					1.02 (0.83-1.27)	0.84
Critical care interventions						
Mechanical ventilation					1.09 (1.01-1.17)	0.03
Pulmonary artery catheter					1.37 (1.12-1.68)	<0.01
Source of infection						
Gastrointestinal					0.93 (0.85-1.03)	0.17
Pneumonia					1.07 (1.00-1.14)	0.04
Skin or soft tissue					0.88 (0.77-1.02)	0.08
Urinary tract					0.83 (0.78-0.89)	<0.01
**Wells 2011**						
Number at risk	132	333				
Age, y	72 ± 13	62 ± 16		<0.01		
Gender (female)	54 (59)	149 (45)	0.85 (0.57-1.29)			
Race						
White	106 (80)	231 (69)	1.80 (1.10-2.93)			
Non-white	26 (20)	102 (31)	0.55 (0.34-0.90)			
Comorbidities						
Diabetes mellitus	54 (14)	111 (33)	1.38 (0.91-2.10)			
COPD	52 (39)	96 (29)	1.60 (1.05-2.45)			
Coronary artery disease	53 (40)	64 (19)	2.82 (1.81-4.39)			

ACE: angiotensin-converting enzyme; AF: atrial fibrillation; APACHE: Acute physiology and Chronic Health Evaluation; ICU: intensive care unit; PAF: paroxysmal atrial fibrillation; SAPS: simplified acute physiology score; SOFA: sequential organ failure assessment.

Data are expressed as mean ±SD, median (interquartile range) or absolute numbers (%).

[a] Age,per 10 years
